# Data normalization considerations for digital tumor dissection

**DOI:** 10.1186/s13059-017-1257-4

**Published:** 2017-07-05

**Authors:** Aaron M. Newman, Andrew J. Gentles, Chih Long Liu, Maximilian Diehn, Ash A. Alizadeh

**Affiliations:** 1 Institute for Stem Cell Biology and Regenerative Medicine, Stanford University, Stanford, California 94305 USA; 2 Division of Oncology, Department of Medicine, Stanford Cancer Institute, Stanford University, Stanford, California 94305 USA; 3 Center for Cancer Systems Biology, Stanford University, Stanford, California 94305 USA; 4 Department of Radiology, Stanford University, Stanford, California 94305 USA; 5 Department of Radiation Oncology, Stanford University, Stanford, California 94305 USA; 6 Stanford Cancer Institute, Stanford University, Stanford, California 94305 USA; 7 Division of Hematology, Department of Medicine, Stanford Cancer Institute, Stanford University, Stanford, California 94305 USA

## Abstract

In a recently published article in *Genome Biology*, Li and colleagues introduced TIMER, a gene expression deconvolution approach for studying tumor-infiltrating leukocytes (TILs) in 23 cancer types profiled by The Cancer Genome Atlas. Methods to characterize TIL biology are increasingly important, and the authors offer several arguments in favor of their strategy. Several of these claims warrant further discussion and highlight the critical importance of data normalization in gene expression deconvolution applications.

Please see related Li et al correspondence: www.dx.doi.org/10.1186/s13059-017-1256-5 and Zheng correspondence: www.dx.doi.org/10.1186/s13059-017-1258-3

**Electronic supplementary material:**

The online version of this article (doi:10.1186/s13059-017-1257-4) contains supplementary material, which is available to authorized users.

Computational approaches for enumerating cell subsets from bulk tissue expression profiles have significant potential for studying tumor cellular ecosystems, including tumor-infiltrating leukocytes (TILs) [1–3]. We therefore read with interest the recent *Genome Biology* article by Li and colleagues in which they introduce TIMER, an in silico method for TIL deconvolution [4]. TIMER relies on prior knowledge of immune signature genes as input and consists of three major steps: (1) gene expression normalization across platforms and sample types; (2) selection of immune signature genes that are negatively correlated with tumor purity; and (3) deconvolution of RNA admixtures using a previously described technique for iterative linear least squares regression (LLSR) [5]. They apply TIMER to the inference of six distinct immune subsets (B cells, CD4 T cells, CD8 T cells, neutrophils, macrophages, and dendritic cells) in The Cancer Genome Atlas (TCGA) bulk tumor expression profiles and investigate links between TIL heterogeneity, tumor genomic features, and survival in 23 cancer types.

Several groups, including ours, have also proposed methods for gene expression deconvolution [1, 3, 5, 6]. We recently described CIBERSORT, an in silico tissue dissection approach that is robust to noise, unknown mixture content, and closely related cell types (collinearity) [6]. Notably, in benchmarking experiments CIBERSORT outperformed other deconvolution methods, including LLSR, and revealed complex associations between 22 distinct immune subsets and outcomes in a pan-cancer meta-analysis [6, 7]. We were therefore surprised by several claims in relation to CIBERSORT.

First, the authors assert that CIBERSORT succumbs to statistical collinearity (i.e., cell subsets with highly correlated expression profiles), leading to biased estimations. Evidence for this argument is primarily based on a simple experiment in which inferred levels of each immune subset were compared by Pearson correlation. After aggregating CIBERSORT results from 22 phenotypes into the same six subsets, the authors compared cross-correlation matrices between TIMER and CIBERSORT on four cancer types. Leukocyte levels estimated by TIMER were almost always positively correlated. According to the authors, positive correlations make intuitive sense because “immune cells work in synergy.” In contrast, the correlations among the six phenotypes estimated by CIBERSORT were largely negative. The authors also observed negative correlations when analyzing more than six cell types with TIMER (i.e., LLSR), stating that negative correlations indicate a technical artifact due to collinearity.

In fact, CIBERSORT mitigates such bias through regularization, as was rigorously demonstrated through a battery of validation experiments [6, 7]. These analyses included an assessment of “deep deconvolution” in which in silico predictions of closely related leukocyte subsets were directly compared against flow cytometry [6]. Moreover, an independent study confirmed that regularization improves the performance of gene expression deconvolution [8].

We were also surprised by the authors’ claim that hematopoietic cell types should generally track together, especially in light of their diverse functions (innate or adaptive, stimulatory or suppressive, etc.) and migration patterns (circulating, tissue-infiltrating, or tissue-resident) [9, 10]. For instance, while specific hematopoietic subsets infiltrating tumors can be positively correlated in a given tumor type [11], the expectation of universally positive correlations does not extend to all tumor types, or to all infiltrating immune cells [12]. Separately, age-related lymphomyeloid lineage skewing of hematopoiesis [13] would be expected to further confound this assumption. Finally, while acute and chronic inflammation can be substrates for tumor initiation, a number of distinct tumor-infiltrating immune cells are known to have either tumor-promoting or anti-tumor properties, and are associated with inverse prognostic correlations with cancer outcomes [9, 14].

We therefore reanalyzed previously published flow cytometry data of leukocyte subsets directly enumerated in peripheral blood mononuclear cells (PBMCs) from healthy donors and in tumor biopsies obtained from patients with lung squamous cell carcinoma (LUSC) [6, 7]. When we quantified each immune subset as a fraction of total leukocyte content, many of the pairwise correlations were negative, as were the mean correlation coefficients (Fig. 1a and b), consistent with CIBERSORT. However, when we instead considered absolute TIL levels in the same lung tumors, most of the correlations were positive (Fig. 1c), likely reflecting differences in tumor purity. Thus, data normalization in solid tumors significantly impacts the assessment of TIL heterogeneity and composition.

**Fig. 1:**
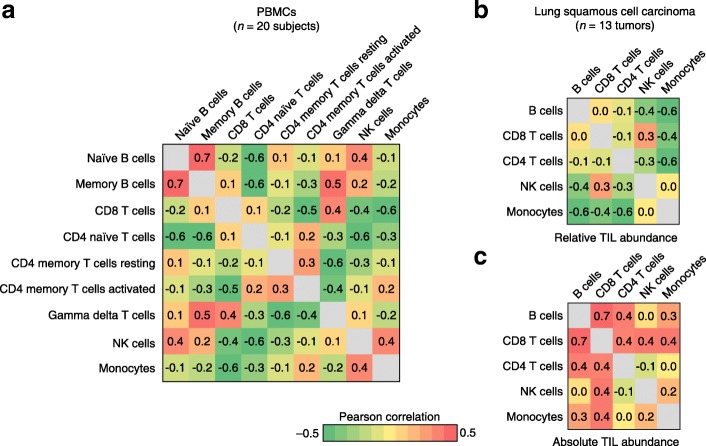
Variable correlation of directly enumerated leukocyte frequencies in lung tumors and blood. **a** All-versus-all Pearson correlation matrix of nine distinct leukocyte subsets profiled by flow cytometry in peripheral blood mononuclear cells (*PBMCs*) from 20 healthy donors [6]. **b** Same as panel **a**, but for five immune subsets profiled by flow cytometry in 13 lung squamous cell carcinoma tumor biopsies [7]. **a**, **b** Leukocyte frequencies were normalized to sum to 1 prior to correlation analysis. **c** Same as panel **b**, except the frequency of each leukocyte subset was expressed as a percentage of viable singlets prior to correlation assessment. *NK* natural killer, *TIL* tumor-infiltrating leukocyte

Given these results, we suspected that tumor purity would explain the discrepancy between TIMER and CIBERSORT. Indeed, after examining the TIMER source code, we found that, unlike most previous deconvolution methods including CIBERSORT, TIMER solves the regression problem without normalizing inferred cell subset frequencies to 1. TIMER results are therefore directly influenced by total leukocyte content, which is inversely correlated with tumor purity across TCGA (Fig. 2a). As a result, all six cell types strongly correlate with total leukocyte abundance in nearly every analyzed tumor type (Fig. 2b), making it difficult to discern the intercellular heterogeneity among the leukocyte subsets that variably infiltrate these tumors (Fig. 2c). When TIMER results were instead normalized in relative space (i.e., summing to 1) for each sample, all mean cross-correlation coefficients were negative (Fig. 2d). The inverse held true for CIBERSORT: mean cross-correlation coefficients became positive when we either (1) omitted the sum-to-1 normalization step, or (2) multiplied the normalized results by a separate estimate of overall immune content (Fig. 2e). While we acknowledge that TIMER estimates were not intended to be analyzed in relative space (as described below), the same reasoning should have been applied by Li et al. to CIBERSORT; that is, CIBERSORT relative abundance estimates should not have been directly compared with absolute leukocyte abundance (as in Table S6 from [4]). Collectively, these data highlight the importance of data normalization in comparing gene expression deconvolution methods.

**Fig. 2:**
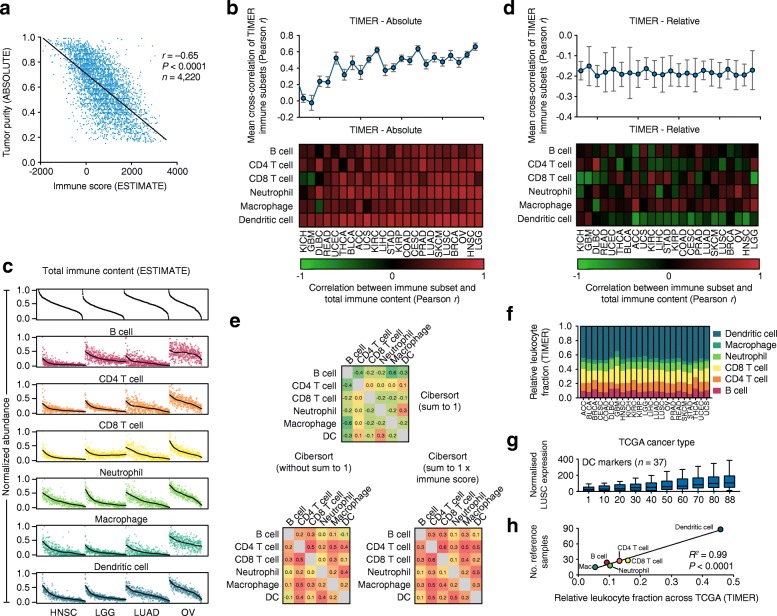
Impact of data normalization on in silico tumor-infiltrating leukocyte profiling. **a** Tumor purity inferred by ABSOLUTE [29] versus immune content inferred by ESTIMATE [30], compared across 11 TCGA (The Cancer Genome Atlas) cancer types (ABSOLUTE data were obtained from [26]). **b** *Bottom* heat map showing Pearson correlations comparing overall leukocyte content, inferred by ESTIMATE, with immune subset abundance, inferred by TIMER, across 23 TCGA tumor types. Cancers are ordered from *left* to *right* by the mean correlation coefficient calculated across the six immune cell types. *Top* mean cross-correlation coefficient of the six immune subsets compared with each other, omitting self-comparisons. Cancer types are vertically aligned, and correlation coefficients are expressed as mean ± SEM. **c** TIMER results are shown for four representative TCGA cancer types, along with immune content inferred by ESTIMATE. Overall leukocyte content and estimates of individual tumor-infiltrating leukocyte (TIL) subsets are normalized from 0 to 1 within each cancer type, and ordered from *left* to *right* by decreasing immune content. *Regression lines* (shown in *black*) were calculated by cubic splines. **d** Same as panel **b**, but after normalizing inferred levels of the six leukocyte subsets to one in each patient. **e** Cross-correlation matrix of CIBERSORT results before and after adjustment by total leukocyte content. Results are shown for lung squamous cell carcinoma (*LUSC*) microarrays profiled by TCGA (*n* = 130 tumor samples). ESTIMATE was used to infer total leukocyte content, denoted *immune score*. **f** Average representation of the six immune subsets inferred by TIMER across 23 TCGA cancer types. **g** Impact of source datasets on tumor gene expression levels following batch correction. Li et al. applied ComBat [17] to merge expression profiles of bulk tumors with a reference database containing six immune cell types with variable representation. Here, the number of dendritic cell (*DC*) samples in the authors’ reference database (*n* = 88) was randomly sampled from 1 to 88 while the remaining immune subsets were left unchanged. For each iteration, ComBat was applied to merge the reference immune profiles with RNA-Seq data from LUSC, which we used as a representative TCGA cancer type (*n* = 555 tumors). The median expression level of each DC marker gene (used in Li et al. and originally obtained from [31]) was determined across the LUSC cohort; markers are represented as medians, quartiles, and 10th and 90th percentiles. **h** Analysis of the number of immune reference samples versus the relative fraction of each immune subset inferred by TIMER across TCGA (colored as in panel **f**)

We and others have previously shown that regression-based gene expression deconvolution can robustly quantify cell-type proportions [5–7, 15, 16]. Therefore, we were surprised by the claim that “levels of different cell types are not comparable” in the output of TIMER [4]. Upon further examination of this output, we found disproportionately high levels of rare dendritic cells (DCs) across all 23 cancer types (approximately 50% by inferred fractional abundance; Fig. 2f), suggesting problems with marker gene selection and/or data normalization. We hypothesized that this result might be due to the authors’ use of ComBat [17] to purge batch effects between two highly distinct sample types: bulk tumors profiled by TCGA and a knowledgebase of purified leukocytes used for signature genes. In support of this hypothesis, we found that the number of DC reference profiles in the knowledge base was strongly correlated with the expression of DC marker genes in normalized tumors (Fig. 2g). Further analysis revealed a strong association between predicted abundance in TCGA (Fig. 2f) and representation in the knowledge base for all six leukocyte subsets (Fig. 2h). Thus, misapplication of ComBat distorted important biological signals that correlate with experimental batches [18], preventing TIMER from estimating cell type proportions.

Separately, we wish to address the claim that CIBERSORT is only applicable to microarray data [2, 4]. While microarray datasets were indeed the focus of our previous studies, this is not an inherent restriction of the deconvolution algorithm itself, which is platform agnostic. In fact, the analytical assumptions made by CIBERSORT are likely to hold for any mixture that can be modeled as a linear sum of its parts and for which an appropriate signature matrix exists. Such mixtures include RNA-Seq datasets, as others have already shown for bulk tumor profiling [19–21], and for single-cell RNA-Seq profiling [22], as well as other genomic features associated with cell lineage [23]. For example, CIBERSORT was recently used to enumerate hematopoietic subsets in bone marrow biopsies from healthy and diseased patients based on genomic patterns of nucleosome accessibility profiled by ATAC-Seq [23], demonstrating its broad applicability.

Finally, in response to this correspondence, Li et al. [24] have made a number of new claims that warrant clarification. In order to comprehensively address these claims, we have included a detailed point-by-point response, including new analyses, in Additional file 1: Figures S1 and S2). We summarize three key points:

1. The authors continue to ignore the significant impact of data normalization on deconvolution results, stating that CIBERSORT produces nonbiological negative correlations mainly due to collinearity. They dismiss the notion that regularization can help combat collinearity (despite significant literature on the topic [25], e.g. ridge regression, and [8]), and offer a flawed analysis to support their claim consisting of synthetic mixture datasets that are improperly defined since the mixed populations do not sum to 100% and are therefore unsuitable for addressing this topic (Additional file 1: Figure S1a and b).
2. Furthermore, Li et al. use a single flow cytometry experiment (Fig. 1a) to argue that closely related immune cell types should be positively correlated in abundance, whether in blood or in tumors. Since the default version of CIBERSORT produced negative correlations for the same cell types when enumerated in solid tumor biopsies, Li et al. claim these results contradict our own experimental data in Fig. 1a and are likely due to collinearity. In making these arguments, Li et al. disregard some fundamental immunological principles governing leukocyte migration patterns (see above and Additional file 1), relevant prior literature (e.g., Fig. 3a in [6]), and the main point of this correspondence (e.g., Fig. 2e). To further illustrate the impact of data normalization on deconvolution results, we extended our CIBERSORT analysis in Fig. 2e to 22 immune subsets (i.e., LM22 [6]) in TCGA LUSC tumors. As expected, the majority of pairwise correlations were positive when relative abundance estimates were scaled by total immune content, including correlations between closely related cell types (e.g., naïve versus memory B cells; Additional file 1: Figure S2). Moreover, we observed no significant association between pairwise correlations of leukocyte estimates in tumors and pairwise correlations of corresponding expression profiles in LM22 (Additional file 1: Figure S2). Therefore, leukocyte behavior is highly complex and unlikely to be distilled into simplistic comigration patterns without significant further investigation, especially without consideration of data normalization.
3. Finally, Li et al. claim that up to 25% of LM22 genes are positively correlated with tumor purity and, as a result, they contend that CIBERSORT’s model is “frequently violated” when applied to tumors. Unfortunately, the authors ignore critical details of the algorithm and the LM22 signature matrix design. They also fail to consider many important factors in the interpretation of their own analyses, including the statistical significance, magnitude, and distribution of correlation coefficients, and the impact of positively correlated LM22 genes on CIBERSORT results. When considering these variables, most of the significant positive correlations are of modest magnitude (e.g., 70% with *r* < 0.2) and only a small minority of LM22 genes are significantly positively correlated with tumor purity (3% with *r* > 0.2, approximately 0% with *r* > 0.4; Additional file 1: Figure S1c). Furthermore, since exclusion of all significantly positively correlated genes from LM22 had virtually no impact on tumor deconvolution performance (Additional file 1), we observed no empirical evidence consistent with the above claim.

In summary, our results address key conclusions in Li et al. [4, 24] and emphasize the importance of data normalization in deconvolution analyses. In particular, deconvolution methods cannot be meaningfully compared without taking normalization differences into account. By focusing on relative measures of TIL content in previous work [7], we avoided the confounding impact of tumor purity [26]. This approach has precedence in prior literature, particularly since many prognostic associations are more robust when defined as ratios of functionally distinct TILs (e.g., CD8 T cells versus Tregs, lymphocytes versus neutrophils, etc.) [7, 27, 28]. Whether absolute or relative measures of TIL abundance better capture tumor immunology in clinical settings remains an important consideration for future studies.

## Additional file

Additional file 1: Detailed response to Li et al., [24]. (DOCX 561 kb)

## Abbreviations

DC: Dendritic cell
LLSR: Linear least squares regression
LUSC: Lung squamous cell carcinoma
PBMC: Peripheral blood mononuclear cell
TCGA: The Cancer Genome Atlas
TIL: Tumor-infiltrating leukocyte
