# Supplementary material for: Data normalization considerations for digital tumor dissection
Source: Genome Biol. 2017 Jul 5;18:128. doi: 10.1186/s13059-017-1257-4 (PMC5498978; doi:10.1186/s13059-017-1257-4)
Supplement: Additional file 1: — Detailed response to Li et al., [24]. (DOCX 561 kb) [file 13059_2017_1257_MOESM1_ESM.docx]

##### Additional file 1

We address three key points from Li et al. [1].

1. Citing no biological evidence, Li et al. [1] state that they “found many non-biological negative correlations between CIBERSORT estimates” on TCGA (The Cancer Genome Atlas) data, and claim this “artifact is, to a large extent, due to “statistical collinearity” rather than data normalization. To support their argument, they conducted an experiment in which two uncorrelated cell types from the LM22 signature matrix (CD8 T cells and neutrophils [2]) were mixed together with added noise (Fig. 1 in [1]). Mixture coefficients were randomly selected from a uniform distribution to ensure the absence of any correlation. Despite this fact, when CIBERSORT was applied to these mixtures, it produced estimations that were negatively correlated. Li et al. concluded that this result demonstrates an artifact of the data normalization approach used by CIBERSORT (i.e., sum to one). They then repeated this experiment using cell types with highly correlated reference profiles (naïve versus memory B cells), and the negative correlation increased. They concluded that data normalization and collinearity will “exaggerate the non-biological negative correlations between the estimated coefficients”.

This analysis suffers from major conceptual and technical flaws. Most importantly, Li et al. base their claims entirely on improperly defined mixtures, in which the sum of the parts is nearly always less than 100% (e.g., 20% CD8 T cells and 40% neutrophils). If such mixtures were instead treated as true mixtures (i.e., sum of parts = 100%), the only formally correct relationship between the two cell types is *by definition* a perfect negative correlation (*r* = –1), assuming that at least some of the randomly drawn coefficients are different. This is not a spurious “non-biological” relationship, since the mixtures created by Li et al. are incomplete and ill-defined. Not only did CIBERSORT correctly identify negative correlations for both cell pairs, but the negative correlation was strongest and, thus, closest to the true relationship between naïve and memory B cells (Fig. 1d in [1]). Therefore, the authors’ own simulation data directly contradict their conclusion.

Furthermore, Li et al. added excessive noise to these pseudo-mixtures, far more than we previously tested [2]. To evaluate how this impacted their results, we compared B cell reference profiles in LM22 before and after the addition of the same amount of noise. The median Spearman correlation was only 0.08, indicating a severe distortion of the reference profiles that is unlikely in practice (Fig. S1a). We repeated the experiment on naïve and memory B cells using the same parameters as Li et al., but with more realistic noise levels when considering technical and biological replicates (Fig. S1a). In both cases, CIBERSORT produced strong negative correlations (Fig. S1b). Notably, these results were comparable to CIBERSORT estimations on CD8 T cells and neutrophils (data not shown). In addition, the above strategy for producing pseudo-mixtures creates highly imbalanced transcriptomes. By simply multiplying the CIBERSORT results (in relative space) by the median expression level of each mixture transcriptome, one can trivially recreate the structure of the original mixing coefficients (“absolute” in Fig. S1b). Datasets used for this analysis can be downloaded from https://cibersort.stanford.edu/timer.php.

2. We included flow cytometry data from various leukocyte subpopulations in peripheral blood mononuclear cells (PBMCs) to illustrate the diversity of cell composition in real biological mixture samples (Fig. 1a). Li et al. [1] have attempted to extrapolate from this single example, arguing (without reference to statistical significance or to the biological literature) that since closely related cell types in one PBMC dataset are positively correlated, they should continue to be positively correlated in other samples, including bulk tumors. Notwithstanding the fact that CIBERSORT estimates were significantly correlated with flow cytometry values for all the cell types cited by Li et al. [2], generalizing in this way is problematic. For example, in a publicly available flow cytometry dataset of PBMCs (SDY311, [www.immport.org](http://www.immport.org/immport-open/public/home/home)), the correlation between naïve and memory B cells is only 0.3, and many related T-cell subsets are negatively or insignificantly correlated (effector memory versus central memory CD4/CD8 T cells are –0.13 and –0.01, respectively). These additional data further support the notion that leukocyte behavior is highly complex and unlikely to be distilled into simplistic comigration patterns without significant further investigation.

Separately, the amount of total immune content in solid tumors/tissues has a considerable impact on pairwise correlations between immune subset frequencies (Fig. 1b and c, Fig. 2e). Failure to consider this critical variable will lead to spurious comparisons analogous to “apples and oranges.”

3. Li et al. [1] claim that the CIBERSORT statistical model assumes that “malignant cells…do not express a significant amount of any of the LM22 genes”, and question whether LM22 genes are truly immune-specific in the context of tumor deconvolution. To address this question, they compared the correlations between LM22 genes and inferred tumor purity levels in TCGA. Based on this analysis, they concluded that up to 25% of LM22 genes are not immune specific (Fig. 2 in [1]), and that the CIBERSORT model is “frequently violated.” Unfortunately, this assessment suffers from several significant shortcomings. First, the authors incorrectly state that LM22 genes were only selected from sorted immune cells, disregarding the fact that we omitted any genes highly expressed in cancer cell lines or enriched in non-hematopoietic tissues [2]. Second, the authors missed the fact that CIBERSORT applies feature selection to the signature matrix, a unique attribute that provides additional noise tolerance by considering only a subset of genes for any given deconvolution. Third, and most important, they ignored the statistical significance of the correlations between LM22 genes and tumor purity, wrongly claiming that any correlation greater than zero is a legitimately positive correlation [3]. Even when taken at face value, the authors’ data show a considerable skew toward negative correlations, and only a small minority of correlation coefficients are >0.2 (Fig. 2 in [1]), which itself is a weak threshold for meaningful covariance in molecular biology—a Pearson *r* of 0.2 corresponds to an *R*2 of only 0.04. Thus, the authors’ conclusion is seemingly at odds with their own data.

To perform this analysis more properly, we calculated Pearson correlation coefficients and corresponding *p* values for 11 cancer types in TCGA with previously inferred tumor purity levels (ABSOLUTE [4], values obtained from [5]). Among genes with significant correlations (Benjamini–Hochberg adjusted *p* value <0.01), only 10% of LM22 genes were positively correlated, with only 3% having coefficients above 0.2 and nearly no genes above 0.4 (Fig. S1c). Furthermore, LM22 genes with negative correlations were 7.5-fold more highly expressed than those with positive correlations (Fig. S1d), indicating that the small minority of positively correlated genes are unlikely to significantly impact deconvolution results. Indeed, removing these genes from LM22 (adjusted *p* < 0.01) had little impact on CIBERSORT results when applied to the microarray version of lung squamous cell carcinoma profiled by TCGA (Spearman correlation across all data points = 0.95).


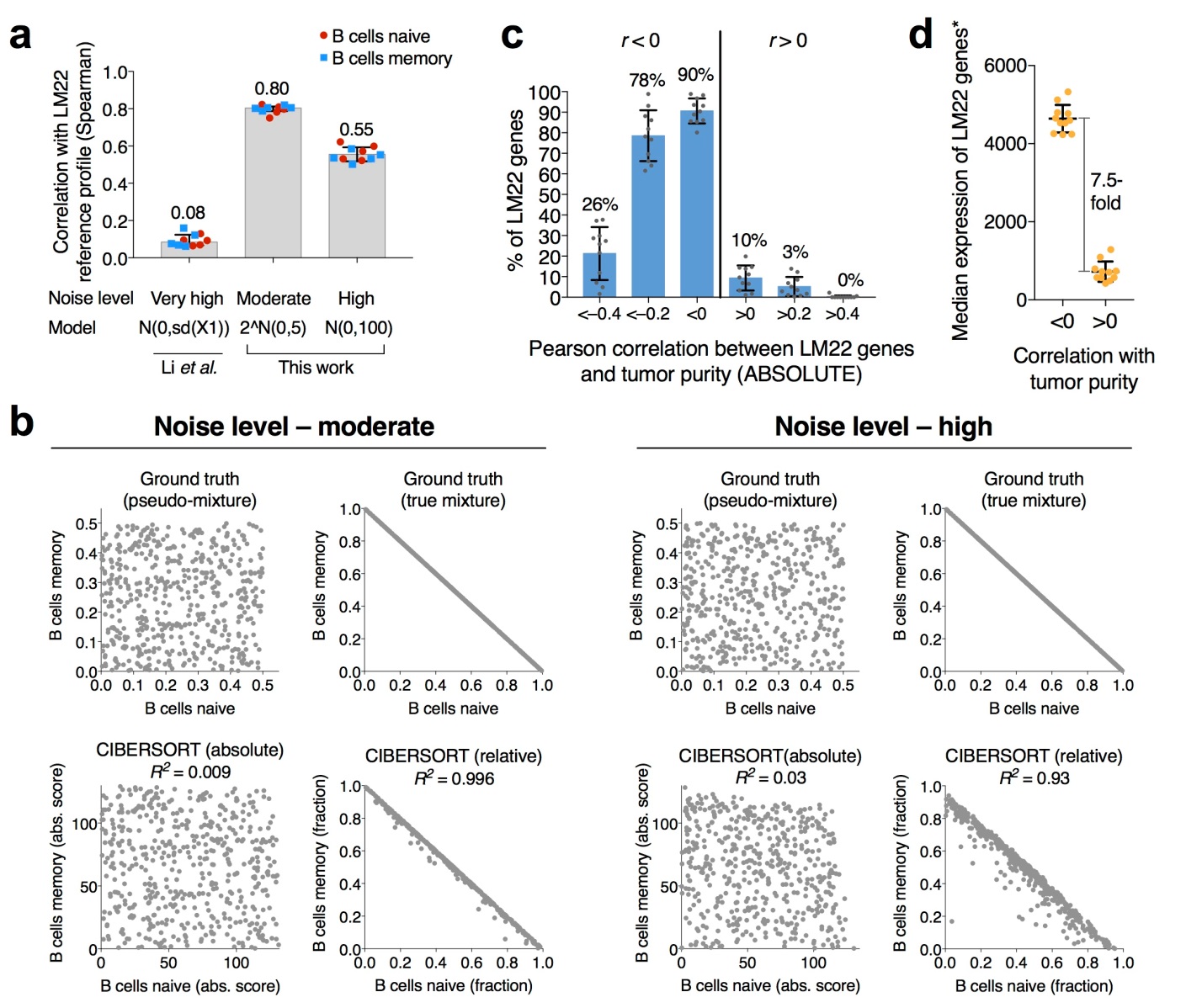


**Fig. S1** Data normalization and statistical considerations, related to Li et al. (2017). **a** Impact of different noise levels on purified B cell reference profiles from LM22. In each case, the original LM22 reference profile was compared to the reference profile after the addition of the indicated noise model. Each noise model was randomly applied five times to the original reference profile, which was first multiplied by 0.25 to approximate the average mixing coefficient used by Li et al. (sampled according to a uniform distribution from 0 to 0.5). Noise levels: “very high” (used in Li et al. [1]); “moderate” (used in “Analysis of multicollinearity” in Newman et al. [2]); “high” (this work only). Medians and interquartile ranges are indicated. **b** Analysis of two-component in silico mixtures consisting of naïve and memory B cell reference profiles, generated as described in Li et al. [1] and in the main text, but using two different noise models described in panel **a**. *Top* the correct mixing proportions of each “pseudo-mixture” are depicted to the *right* (i.e., “true mixture”). *Bottom* CIBERSORT results for the analysis of the pseudo-mixture indicated above, with default CIBERSORT results shown on the *right* (relative space) and default results multiplied by the median expression level of each pseudo-mixture sample shown on the *left* (absolute space). Note that CIBERSORT was run without quantile normalization to avoid perturbation of the pseudo-mixtures. **c** Analysis of the correlation between LM22 gene expression and tumor purity, across 11 TCGA (The Cancer Genome Atlas) cancer types with previously inferred purity levels [5]. Shown are percentages of LM22 genes within different correlation bins across the 11 cancer types. Means and standard deviations are indicated by *bar heights* and *error bars*, respectively. The median percentage of LM22 genes within each correlation bin is provided above the corresponding bar. **d** Analysis of LM22 gene expression in relation to LM22 genes that are positively or negatively correlated with tumor purity from panel **c**. The a*sterisk* indicates that, for each LM22 gene, only the highest expression level across the 22 leukocyte subsets was considered. Only genes significantly correlated with tumor purity (Benjamini–Hochberg adjusted *p* value <0.01) were analyzed in panels **c** and **d**. All experiments were performed with the R version of CIBERSORT (version 1.04)

#####
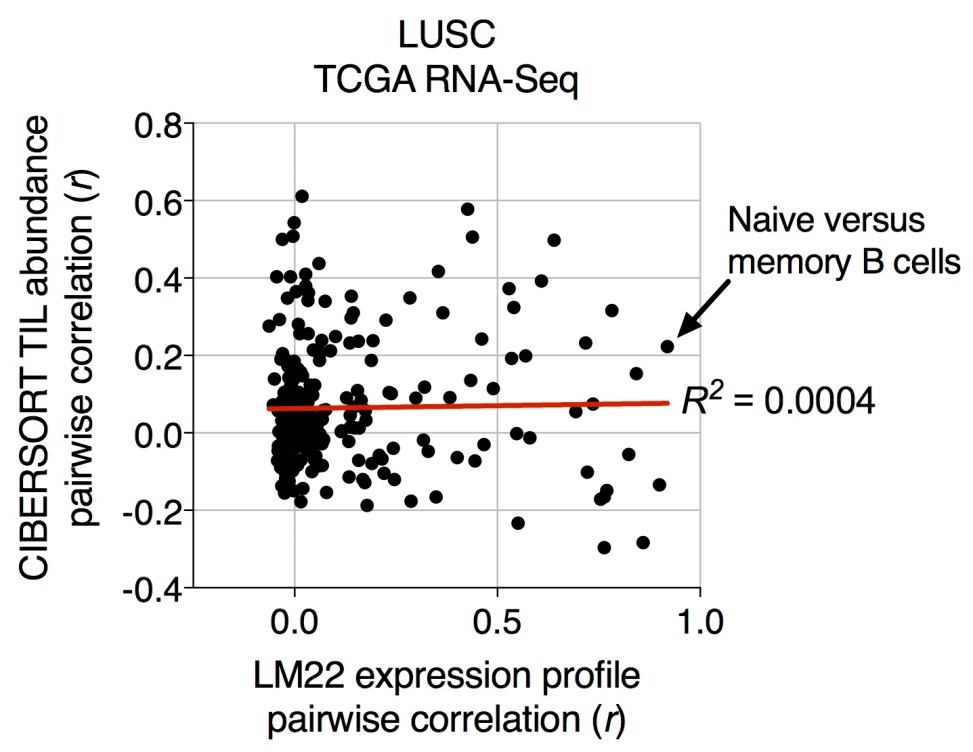


##### Fig. S2. Relationship between pairwise correlations in tumor-infiltrating leukocyte (*TIL*) abundance and pairwise correlations in corresponding immune reference profiles, related to Li et al. (2017). Scatterplot showing pairwise Pearson correlations between LM22 immune reference profiles (*x* axis*, n* = 231 unique pairings, excluding self-comparisons) versus pairwise Pearson correlations for the corresponding cell subsets enumerated by CIBERSORT in TCGA (The Cancer Genome Atlas) lung squamous cell carcinoma (*LUSC*) tumors (*y* axis, *n* = 555 tumors profiled by RNA-Seq). Relative abundance estimates for the latter were multiplied by total immune scores inferred by ESTIMATE [6] (a constant was added to all immune scores to remove negative values). Notably, these scores were calculated from immune-associated genes and should not be significantly confounded by tumor or stromal content (e.g., Fig. 1 in [6]). To verify this result, we repeated this analysis using immune content inferred by DNA methylation signatures (available from www.synapse.org; [4]). This orthogonal measure of total immune content yielded comparable results (*R2* = 0.0005)

##### Additional file 1: References

1. Li B, Liu JS, Liu XS. Revisit linear regression based deconvolution methods for tumor gene expression data. Genome Biol. 2017. doi:10.1186/s13059-017-1256-5.

2. Newman AM, Liu CL, Green MR, Gentles AJ, Feng W, Xu Y, et al. Robust enumeration of cell subsets from tissue expression profiles. Nat Methods. 2015;12:453–7.

3. Altman N, Krzywinski M. Points of significance: association, correlation and causation. Nat Methods. 2015;12:899–900.

4. Carter SL, Cibulskis K, Helman E, McKenna A, Shen H, Zack T, et al. Absolute quantification of somatic DNA alterations in human cancer. Nat Biotechnol. 2012;30:413–21.

5. Aran D, Sirota M, Butte AJ. Systematic pan-cancer analysis of tumour purity. Nat Commun. 2015;6:8971.

6. Yoshihara K, Shahmoradgoli M, Martinez E, Vegesna R, Kim H, Torres-Garcia W, et al. Inferring tumour purity and stromal and immune cell admixture from expression data. Nat Commun. 2013;4:2612.
